# Random and Natural Non-Coding RNA Have Similar Structural Motif Patterns but Differ in Bulge, Loop, and Bond Counts

**DOI:** 10.3390/life13030708

**Published:** 2023-03-06

**Authors:** Fatme Ghaddar, Kamaludin Dingle

**Affiliations:** 1 Department of Computer Science, Gulf University for Science and Technology, Hawally 32093, Kuwait; 2 Centre for Applied Mathematics and Bioinformatics (CAMB), Department of Mathematics and Natural Sciences, Gulf University for Science and Technology, Hawally 32093, Kuwait; 3 Department of Computing and Mathematical Sciences, California Institute of Technology, Pasadena, CA 91125, USA

**Keywords:** RNA, evolution, genotype–phenotype maps, phenotype bias, ‘junk’ DNA

## Abstract

An important question in evolutionary biology is whether (and in what ways) genotype–phenotype (GP) map biases can influence evolutionary trajectories. Untangling the relative roles of natural selection and biases (and other factors) in shaping phenotypes can be difficult. Because the RNA secondary structure (SS) can be analyzed in detail mathematically and computationally, is biologically relevant, and a wealth of bioinformatic data are available, it offers a good model system for studying the role of bias. For quite short RNA (length L≤126), it has recently been shown that natural and random RNA types are structurally very similar, suggesting that bias strongly constrains evolutionary dynamics. Here, we extend these results with emphasis on much larger RNA with lengths up to 3000 nucleotides. By examining both abstract shapes and structural motif frequencies (i.e., the number of helices, bonds, bulges, junctions, and loops), we find that large natural and random structures are also very similar, especially when contrasted to typical structures sampled from the spaces of all possible RNA structures. Our motif frequency study yields another result, where the frequencies of different motifs can be used in machine learning algorithms to classify random and natural RNA with high accuracy, especially for longer RNA (e.g., ROC AUC 0.86 for *L* = 1000). The most important motifs for classification are the number of bulges, loops, and bonds. This finding may be useful in using SS to detect candidates for functional RNA within ‘junk’ DNA regions.

## 1. Introduction

Within evolutionary biology, a long-standing debate has centered on whether, and in what ways, development and biases in the genotype–phenotype (GP) map can be a directive force in evolution [1,2]. In principle, many factors, including selection, historical contingency [3,4], random drift, and biases arising from non-isotropic phenotype variations [5], could have important roles in shaping evolutionary outcomes [6]. Untangling the relative contributions of each factor is difficult, leading to a protracted debate; clear data to adjudicate between the various positions has been lacking. Despite these challenges, many studies point to biases in GP maps and development [7,8,9], and, similarly, mutation biases [10,11,12], shaping evolutionary trajectories.

One system that can be used to shed light on the question of bias in evolution is the RNA sequence-to-structure map, where the secondary structure (SS) is taken to be a phenotype describing the pattern of nucleotide-base bonding. This map is both computationally and mathematically tractable; for example, algorithms exist for predicting the SS directly from sequences [13,14]. At the same time, it is a biologically relevant system because RNA is a versatile molecule that fulfills many diverse functions in living organisms, such as information transfer, catalysis, sensing, and regulation. Moreover, it is well known that the RNA SS is important for functional RNA [15,16,17], and even for messenger RNA [18,19,20]. The SS is also an important determinant of RNA tertiary structures [21]. For these reasons, the RNA SS has been studied extensively for many years to elucidate the properties of the GP map and as a model system to study evolution [22,23,24,25,26,27,28,29,30].

It has been known for many years that the RNA sequence-to-structure map is biased in the sense that some SSs disproportionately have many sequences underlying them [23,25]. In other words, there is an exponential variation in the probability of obtaining different SSs upon the uniform random sampling of RNA sequences, with a small proportion of possible SSs accounting for a large fraction of all possible sequences, while many SSs only have very few sequences assigned to them.

Dingle et al. [31] studied the RNA SS map in the context of the role of bias in evolutionary dynamics. Via computational analysis, they compared natural non-coding RNA of lengths L≤126 nucleotide(s) (nt) with randomly generated RNA in terms of the distribution of *neutral set sizes* (i.e., the number of sequences per SS). They found that the distributions were surprisingly similar. The authors mathematically inferred the distribution of neutral set sizes, which would appear from uniform sampling over SS phenotypes (what they called *P-sampling*), and compared it to the observed computationally generated distribution from genotype sampling (what they called *G-sampling*), as well as the distribution from natural data. They also studied other properties of SSs, again finding similarities between natural and random SSs. Moreover, the properties of natural RNAs were very different from those of a typical SS of the same length within the full space of all possible SSs. Their main conclusion was that the GP structure appears to strongly constrain which SSs are found in nature. Later, Dingle et al. [32] extended the earlier study (still using ncRNA with short lengths), by comparing natural and random RNA SS-abstracted shapes, not just some of the properties of the SS. Their main conclusion was that the shapes of molecules in nature were very similar to shapes derived from the random sampling of genotypes, and that the shape frequencies were close to what would be expected from random sampling. The fact that the authors could predict natural shape abundances merely from computer simulations is quite striking. This suggests that the GP map itself is a dominant factor in determining the repertoire and frequency of extant non-coding RNA shapes in nature.

A limitation of these earlier works is that only quite small RNAs of lengths L≤126 nt were studied, whereas many natural RNAs were far larger, leaving the generalization of the earlier findings to larger—and biologically more interesting—RNA an open question. Much earlier, Fontana et al. [22] pioneered comparisons of natural and random RNAs, finding significant similarities, but their analyses were limited by the datasets available at the time. Here, we extend earlier studies by investigating two questions: From the theoretical side, we studied much larger non-coding RNA of up to L=3000 nt to determine the role of GP map bias in defining the existing repertoire of RNA using structural motifs in addition to RNA shapes.

From the practical side, we ask if motif counts can be used to distinguish (or classify) natural vs. random RNA, which may be useful in the context of detecting functional RNA in non-coding regions of the genome [33,34]. This is an important question because while a large fraction of the human genome is transcribed, less than 2% of the genome are protein-coding regions [35]. The function of the other RNA transcripts—or the lack thereof—is a subject of intense research. Furthermore, recent results point to the possibility of uncovering the functions of RNA transcripts that have been previously only considered ‘junk’ regions of DNA, with possible functions including stimulation of the immune system during viral infection, in tumor cells, or as a consequence of autoimmune disease [36,37]. Further, with new high-throughput methods to perform the full RNA sequencing of a cell, many new RNA transcripts are rapidly being discovered [38]. If functional RNA can be detected with very simple methods, such as counting structural motifs, then this would aid in these current research directions.

## 2. Results

### 2.1. Abstract RNA Shapes

**Figure 1:**
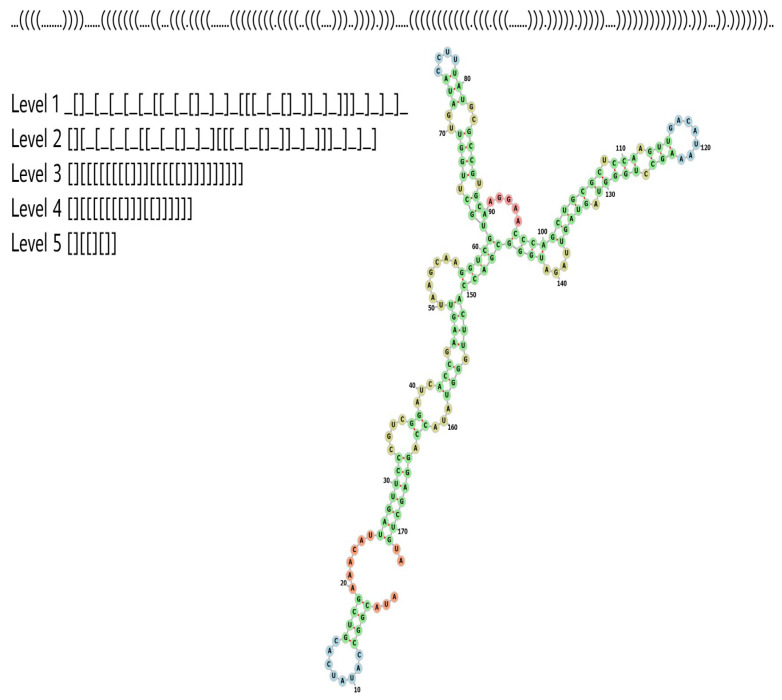
*Sepia pharaonis* 5S ribosomal RNA, abstract shape illustration (length is L=173). The dot-bracket and abstracted shapes at higher levels are displayed, corresponding to progressively more coarse-grained shapes.

In studies on molecular evolution and bioinformatics, it is common practice to represent RNA SS in dot-bracket form, where a dot represents an unpaired nt base, and a bracket a paired base. Left and right brackets must, therefore, match up; see Figure 1.

However, studying dot-bracket RNA has some drawbacks: Firstly, a natural RNA, such as the *Sepia pharaonis* 5S ribosomal RNA depicted in Figure 1, may (in nature) show small variations in length and structural properties, such that an RNA can have slightly different dot-bracket structures. In contrast, the dot-bracket representation defines each different dot-bracket structure as a different RNA phenotype. Hence, this type of representation can be seen as perhaps overly detailed for some purposes. Secondly, because of the large variety of different dot-bracket RNAs, obtaining statistics about the frequency of a given SS from a database is difficult, because a SS must be found many times to deduce an accurate frequency. This is especially onerous when working with natural databases, which may only have small numbers of samples of each length.

To begin our analysis, following reference [32] (see also [39,40]), we used the RNA shapes [41,42] method. According to this method, an RNA dot-bracket SS can be abstracted to one of five levels, by increasing abstraction and ignoring details, such as the loop lengths, and including broad-shaped features. Figure 1 illustrates these levels for the *Sepia pharaonis* 5S ribosomal RNA. The choice of the level is a balance between being detailed enough to capture important structural aspects, but not too detailed, such that for a given dataset, many shapes are possible, and very few repeated shapes are found, making it impossible to obtain reliable frequency/probability values. In this current investigation, considering our datasets, we will use level 5 throughout this work.

### 2.2. Nature Uses High-Frequency Shapes

To compare shape frequencies for natural and random RNA, we first computationally generate random RNA sequences, then predict their corresponding dot-bracket SSs using the popular RNA Vienna package [43]. Then, dot-bracket SSs are converted into abstract shapes, as described above. To compare to natural SS, we took natural RNA sequences from the RNAcentral [44] database, which is a well-populated database of non-coding RNA, and predict the SS using the RNA Vienna package. Here, we study lengths *L* = 100, 200, 300, and 400 for both random and natural sequences. The probability (or frequency) of each phenotype shape *p* is calculated as the fraction of all shapes, which have shape *p*. See Methods for more details.

**Figure 2:**
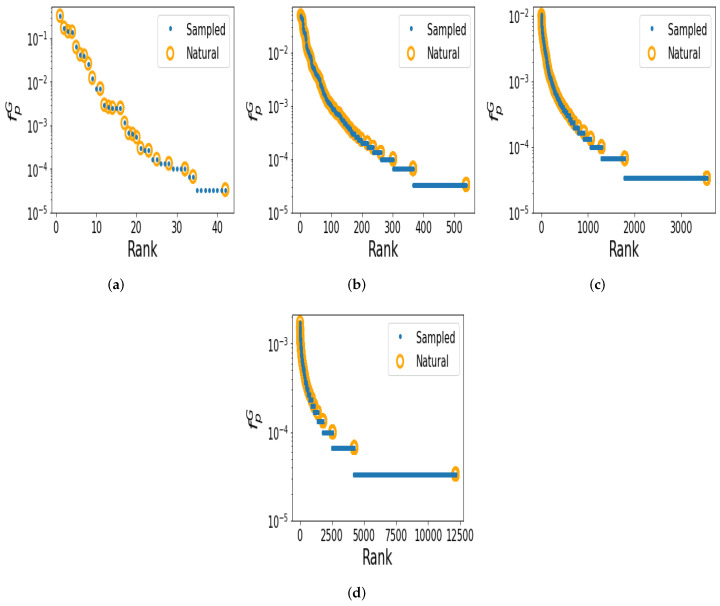
Nature selects frequent structures. Rank plots depicting the probability $f_{p}^{G}$ of random (sampled) in blue and natural RNA secondary structure (SS) abstract shapes in yellow. The shapes in nature tend to be those of high probability (i.e., high frequency). (**a**) Length L= 100 nucleotides (nt); (**b**) L= 200 nt; (**c**) L= 300 nt; (**d**) L= 400 nt.

Using the derived frequencies, we can make rank plots that have the frequency for each shape on the *y*-axis, and the rank of the shape on the *x*-axis. The highest frequency corresponds to rank 1, the second highest frequency to rank 2, etc. The rank plots are shown in Figure 2, where blue dots indicate random shapes, and yellow circles represent the natural shapes that appear in the RNAcentral database. The shapes in nature tend to have higher frequencies.

We generated 30,000 random sequences for each of the lengths L=100, 200, 300, and 400. The unique shape numbers found from sampling 30000 random sequences for each length were 42 (*L* = 100), 538 (*L* = 200), 3551 (*L* = 300), and 12,149 (*L* = 400). From the database of natural sequences, the natural sequence numbers we used were 20,223 (*L* = 100), 37,474 (*L* = 200), 19,496 (*L* = 300), and 34,858 (*L* = 400). The unique resulting natural shape numbers that came from the natural database sequence were 35 (*L* = 100), 575 (*L* = 200), 2494 (*L* = 300), and 8738 (*L* = 400).

It is interesting to calculate the fraction of natural shapes that appeared the random sampling, i.e., the fraction of the unique natural shapes found by sampling random sequences. The fractions of unique natural shapes found by random sampling for each length, respectively, were 34/35 (97%), 397/575 (69%), 1679/2494 (63%), and 4111/8738 (47%). It is interesting that in these cases, most of the shapes in nature were found by modest samplings for L=100, 200, and 300, and nearly half for L=400, suggesting that nature mainly utilizes RNA shapes, which are high frequency and, hence, easy to ‘find’; low frequency shapes are not (or rarely) found in nature (see also reference [45] for computational work in a similar vein).

To help quantify how strong the effect of the phenotype bias is, we can use an estimate of how many shapes actually exist for each length we studied. If there are not many possible shapes for a length *L* RNA, then it is not very surprising that modest random sampling finds most of the natural shapes. If there are very many possible shapes, then it is highly unlikely that the relatively modest numbers of natural and random sequences should have shapes that coincide, unless the bias is very strong. Nebel and Scheid [46] made approximate analytic estimates of the number of shapes of length *L* (while ignoring the fact that some shapes may not in fact be designable). For level 5, the number of shapes ($s_{5_{L}}$) is $s_{5_{L}}\approx 2.44\times 1.32^{n}\times n^{-\frac{3}{2}}$, where we have taken the results pertaining to a minimum hairpin length of 3, and a ‘min’ ladder length (which applies to the Vienna folding package). Thus, both are exponential in length *L*. From these equations, we have $s_{5_{100}}$≈$10^{9}$, $s_{5_{200}}$≈$10^{21}$, $s_{5_{300}}$≈$10^{33}$, $s_{5_{400}}$≈$10^{45}$. So we see that the spaces of possible shapes are astronomical for these lengths. As only a tiny fraction of these shapes were actually found by sampling, we can infer that the bias must be very strong for these large RNAs, and that nature tends to only use high-frequency shapes.

### 2.3. Shape Abundance Can Be Predicted from Random Sampling

**Figure 3:**
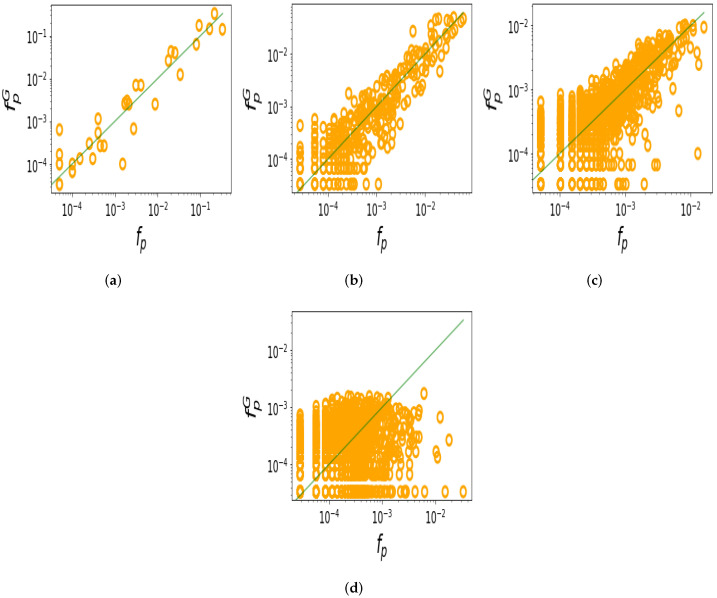
The probability $f_{p}^{G}$ of RNA shapes from random sampling positively correlates with the probability $f_{p}$ of shapes in nature. (**a**) *L* = 100, linear correlation of log probability *r* = 0.94; (**b**) *L* = 200, *r* = 0.89; (**c**) *L* = 300, *r* = 0.79; (**d**) *L* = 400, *r* = 0.44 (all *p*-values $\ll 10^{-6}$).

Next, we compare the probability $f_{p}^{G}$ with which RNA shapes appear in random sampling with the probability $f_{p}$ that they appear in the database. This is immediately related to the preceding investigation, but looking at correlations between probabilities (or equivalently, the frequencies) is more nuanced than simply looking at whether high-frequency shapes appear or not. So, in Figure 3, we show the same data as Figure 2, but now as correlation plots. For *L* = 100, 200, and 300, there is a clear positive correlation between the probability $f_{p}^{G}$ in which shapes appear in randomly generated sequences, and the probability $f_{p}$ with which they appear in nature (linear correlations of log probabilities are *r* = 0.94, *r* = 0.89, *r* = 0.79, respectively, all with *p*-values $\ll 10^{-6})$. For *L* = 400, the correlation is weak (*r* = 0.44, *p*-value $\ll 10^{-6}$), which is likely due to the very noisy frequency data. The total number of possible shapes increases exponentially with length; hence, much more data are required to obtain decent statistics for longer lengths. We can conclude that not only does nature typically use high-frequency shapes, but the shape frequencies in nature tend to be similar to those from random sampling. Note that if natural structures were uniformly chosen from the spaces of possible phenotypes (‘P-sampling’ [31]), then the natural frequencies would be close to uniform and not correlate with the frequencies of the sampled structures.

### 2.4. Studying Structural Motif Frequencies for Larger RNA

As seen above, for lengths beyond ∼300 nt, even at the abstract shape level 5, having enough data available to estimate the shape frequencies to high accuracies is challenging. Hence in order to study much larger RNA, we take a different approach. We will compare the computationally folded natural and random structures in terms of fairly easy-to-calculate structural feature motifs, namely the number of helices, bonds, loops, junctions, and bulges. That is, for each RNA dot-bracket SS, we will count these motifs and plot them for natural and random RNAs with lengths of 50≤L≤3000 (Methods). In this manner, we can see if, for larger RNA, the structural motifs of natural and random SS are similar or not. While the motifs and full RNA shapes are not equivalent, the number of helices, bonds, loops, junctions, and bulges, will be related to the overall shapes. See the Appendix B for more on this relation.

**Figure 4:**
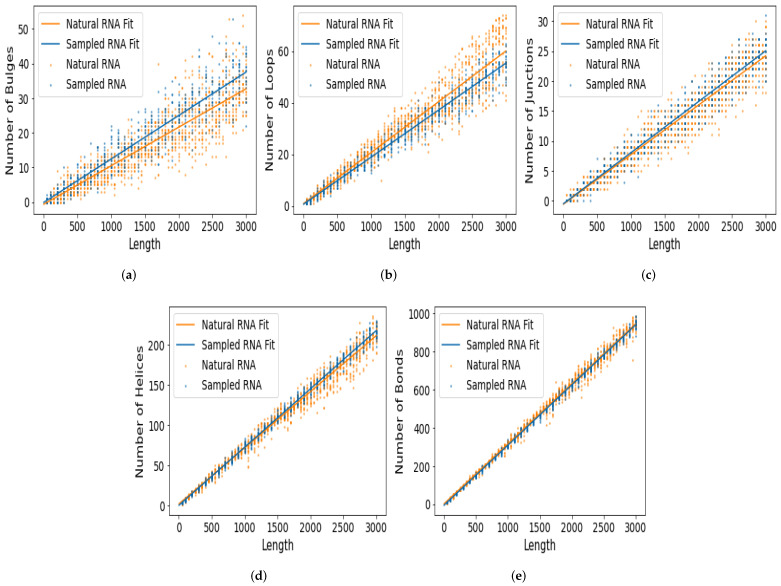
Natural RNA up to *L* = 3000 has a similar number of bulges, loops, junctions, helices, and bonds as randomly sampled RNA. The frequencies of bulges and loops appear to differ the most between natural and randomly sampled RNA.

**Figure 5:**
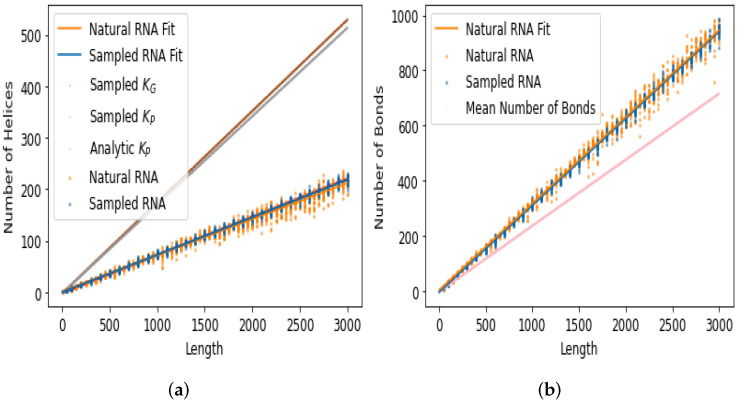
The frequency of (**a**) helices and (**b**) bonds observed in natural and random sampling are both very different from those expected from uniform sampling over phenotype SS (P-sampling). *Sampled* $K_{G}$ denotes the mean number of helices obtained from random genotype sequence sampling (G-sampling), and *Sampled* $K_{P}$ denotes the mean number of helices expected for uniform random sampling over all possible structures (P-sampling) [31]. *Analytic* $K_{P}$ denotes the analytic estimated mean number of helices expected for uniform random sampling over all possible structures [47]. The estimated mean number of bonds is also taken from [47].

Figure 4 shows the motif frequency count for each of the five motifs. The results show that the counts for natural and random RNA are quite similar. The bulges and loops exhibit the most significant difference, with the most divergent lines of best fit for the natural and random data. However, the differences are still relatively small. For junctions, helices, and bonds, the lines of best fit for natural and random RNA are very close. In the case of the frequency of bonds and frequency of helices, we also plot in Figure 5 analytic predictions [47] and a computational fit [31] for the expected frequency, obtained from P-sampling (i.e., uniform sampling over all possible SS). As is clear from the figure, neither the natural data nor the random data are similar to the P-sampled values. In particular, the natural and random data are far more similar to each other than they are to the P-sampled frequencies.

The linear fits for the motif counts, as functions of length *L* presented in Figure 4, are given in Table 1. The linear fits are m=aL+b, where *m* is the frequency of motif *m* and *a* and *b* are the slope and intercept of the fit. Table 2 additionally gives the 95% confidence intervals for the fitting parameters based on bootstrap sampling.

As a side comment, in reference [31], the authors used the neutral network size estimator [48] to compare the neutral set sizes (number of sequences per SS) of natural and random RNA of L≤126. On trying to use this neutral network size estimator for much larger *L*, we found that it is not suitable due to increased computational costs and an increasing failure rate in terms of the fraction of sequences for which the algorithm fails to converge.

As noted by earlier researchers [49], because the GC content of natural RNA sequences is often biased away from a uniform nucleotide composition value, it is important to check that any observed differences between natural and random RNA are not merely due to differences in the nucleotide composition. Hence, we also checked that the observed differences in the motif counts persist when using ‘scrambled’ natural RNA sequences (i.e., randomly permuted natural sequences) instead of uniformly random sequences. The same overall pattern of observations persists, as can be seen in Appendix C. This suggests that GC bias alone does not cause differences between the natural and random samples that we observe.

### 2.5. Biological Functions of Some High and Low-Frequency Shapes

**Figure A1:**
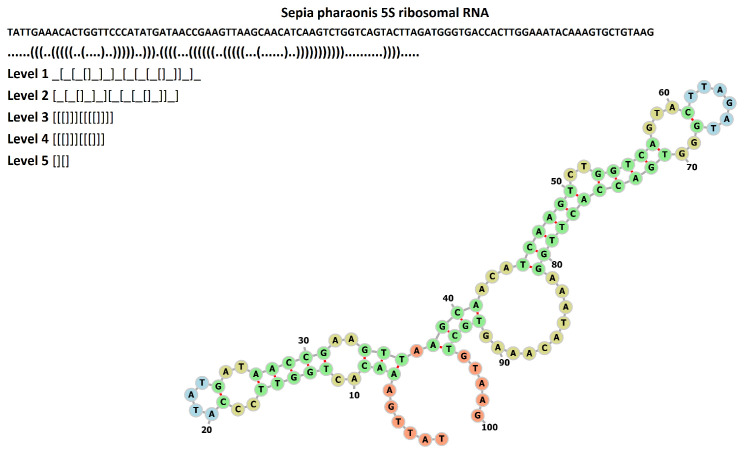
The figure shows the abstract and graphical shape illustrations for *Sepia pharaonis* 5S ribosomal RNA (length is *L* = 100), which has the most frequent random abstract shape of [][] in level 5. The dot-bracket and abstracted shapes at all levels are displayed, corresponding to progressively more coarse-grained shapes.

We now look at shapes with high and low frequencies in terms of what biological functions they perform. If an RNA has a high frequency, then it can be found by only modest sampling. This is interesting from an evolutionary perspective because it suggests that natural selection does not have to ‘work hard’ in order to shape the RNA. On the other hand, it would be interesting to see if some natural RNAs have very low frequencies, which would suggest that selection had to ‘work hard’ to form that shape. We extracted the highest frequency shape, which appeared in random sampling. We then searched for any molecules in the natural RNA samples from the RNAcentral database, which had the same shapes. The most frequent random abstract shapes for the lengths *L* = 100, 200, 300, and 400 are [][], [[][][]], [[][][]][], and [][[][][][][]]. In the Supplementary Information, Excel sheets are given, which give lists of the RNA names and frequencies of each frequency shape. In Appendix A, Figure A1 shows an example RNA with shape [][]. Here, we just give the names of the RNAs that had the highest frequencies, which were *Sepia pharaonis 5S ribosomal RNA*, *uncultured bacterium partial 16S ribosomal RNA*, *Hevea brasiliensis miscellaneous RNA*, and *uncultured bacterium bacterial SSU rRNA*, for *L* = 100, 200, 300, and 400, respectively.

There is more than one random shape that occurs only once for each length; hence, there are numerous ‘least frequent’ shapes in the random samples. Some examples are the random abstract shape [][[][]][][] with *L* = 100 appeared only once in random sampling; the molecule labeled as the *unclassified sequence of pemK RNA* was found to have the same shape. Among the lowest frequency shapes from the random samples for *L* = 200 is [[[][]][[][]]][][][], no natural RNAs were found to have this shape. One of the lowest frequency shapes from the random sampling for *L* = 300 was [][][][[][[][]][][]][][], and this had one occurrence in the natural data, namely *uncultured bacterium partial 16S ribosomal RNA*. For *L* = 400, one of the lowest frequency shapes was [[[][][]][[][]]][[][][]][], but this was not found among the natural RNA samples.

## 3. Classifying Natural and Random RNA Using Motif Counts

### 3.1. Can We Use Motif Frequency to Detect Functional RNA?

The preceding sections showed that natural and random RNAs are overall very similar, especially when compared to the full space of possible RNA SS. However, there are some differences in the motif counts between natural and random RNAs. Given that we saw some small differences in the motif frequencies, here, we will attempt to distinguish or classify natural and random RNA using RNA SS motif counts. One potential application of this would be in detecting functional RNA in supposed ‘junk’ non-coding regions of the genome, as discussed in the Introduction.

Several studies attempted to identify functional RNA or classify different types of RNA. Noteworthy examples include the following. Rivas and Eddy [49] attempted to distinguish between random and natural RNAs, similar to what we considered here. They initially found that SS can be used to distinguish between natural and random RNAs, but then this ability to distinguish disappeared after adjusting for the GC content. Further, they reported that the calculated thermal stability of most functional RNA SS is not sufficiently different from the predicted stability of a random sequence to detect functional RNA. Carter et al. [50] concluded that using free energy folding values improved function detection beyond just sequence motifs. Bonnet et al. [51] showed that microRNA had lower folding free energies than random sequences, but reported that they did not find a good general method to distinguish between natural and random RNAs because their method did not work on, e.g., tRNA. Later, Washietl et al. [52] distinguished between natural and random RNAs using the thermal stability of folds. To classify ncRNAs of different organelle genomes, Wu et al. [53] used a machine learning approach involving sequence information and frequency counts of the stems, junctions, hairpin loops, bulge loops, interior loops, and the total loops with more than three bases. Their work correlates with ours in that they used structural motif counts, but differs in that we did not attempt to distinguish between RNA derived from different organelles. More recently, Sutanto and Turcotte [54] employed machine learning and structural aspects to classify sequences into specific ncRNA classes. Agai, this study is related to our current question but differs in that we did not attempt to distinguish between different types of natural ncRNA classes.

### 3.2. Classifying RNA

We attempted to classify natural and random RNA. The datasets have five dimensions, where the features (variables) are the frequency counts of bonds, helices, loops, bulges, and junctions on each SS. There are many algorithms in machine learning that can be used for classification. At first, we used *k*-nearest neighbor (kNN), which is a very common and versatile learning algorithm. We performed five-fold cross-validation; classification accuracy was quantified in terms of ROC AUC. Bootstrap sampling was used to obtain 95% confidence intervals for the ROC AUC values. Note that for binary classification, an ROC AUC value of ∼0.5 indicates very poor classification, no better than guessing classes. Higher values indicate better performances, with 1.0 denoting perfect classification ability.

To experiment, we used the following datasets: L=100 with 30,000 random and 20,223 natural RNA SS, L=400 with 30,000 random and 34,858 natural RNA SS, and L=1000 with 1000 random and 4836 natural RNA SS. (Note that counting motifs for very large RNA becomes computationally taxing, hence the reduced sample size for L=1000.) The results are presented in Table 3, and we see that for longer RNA, the classification accuracy is quite high, at around 0.86. The fact that the classification performance is not as high for shorter RNA sequences is expected from Figure 4, where we saw that the natural and random lines of best fits had slightly different slopes, such that for longer RNA, they were more clearly distinguishable. Hence, we can expect that classification accuracy is lower for shorter RNA and higher for longer RNA.

Due to the importance of checking that predictive accuracy is not merely due to a different GC content value [49], for L=1000, we further created a ‘scrambled’ dataset, which was made by randomly permuting natural RNA sequences to maintain the same GC content as the natural data. This adjustment for GC content barely lowered the classification performance, as shown in Table 3.

The kNN method has the benefit of being able to handle arbitrary patterns in data, provided that enough data are available. However, it does not provide an indication of which features (variables) are important in distinguishing the groups. As a different machine learning perspective, we also implemented the partial least squares discriminant analysis (PLSDA) method, which is a linear method that also yields *variable importance*, i.e., a signed value indicating which features (variables) are the most important in distinguishing the groups (larger magnitudes indicate greater importances). The PLSDA method gave ROC AUC values that are similar to—but slightly lower than—the kNN method, as indicated in Table 3.

**Figure 6:**
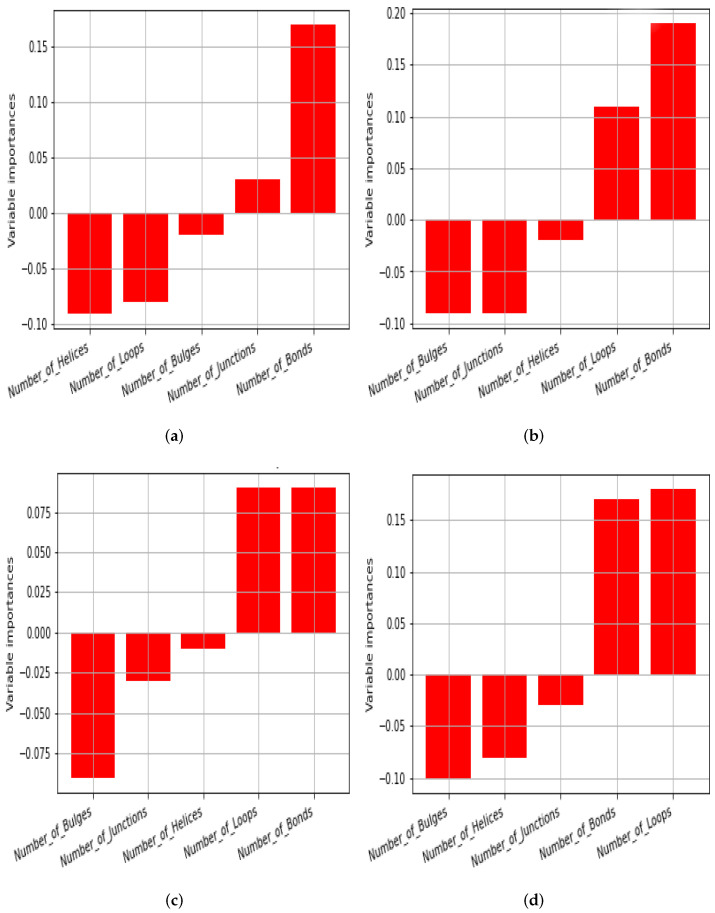
Variable importance plots for different lengths of RNA. (**a**) Length *L* = 100 natural versus random RNA samples, ROC AUC is 0.70 when evaluated using PLSDA (kNN gives 0.72). Bonds are the most important variable. (**b**) Length *L* = 400 natural versus random RNA samples, ROC AUC is 0.78 when evaluated using PLSDA (kNN gives 0.81). Bonds and loops are the most important variables. (**c**) *L* = 1000, with ROC AUC 0.83 for PLSDA (kNN gives 0.86). Loops, bonds, and bulges are the most important variables. (**d**) After adjusting the GC content, ROC AUC is 0.83 using PLSDA (kNN, ROC area is 0.86). Loops, bonds, and bulges are the most important variables.

Figure 6 shows a plot of the variable importance for the natural vs. random data and the natural vs. ‘scrambled’ data. The figure indicates that the number of bonds is the most important for the small RNAs of L=100, the bond and loop numbers for L=400, and that for the bulges, loops, and bond counts are the most important for L=1000. The helix and junction numbers are relatively unimportant in distinguishing natural and random RNA. Note that in Figure 4 it is visually clear that bulges and loops show the largest differences between natural and random SS, so it follows that they should appear with large variable importance. Regarding why the bond count also seems to be significant here (even though it may not be apparent from Figure 4), the reason is unclear. It is possible that there are some types of multivariate interactions between the motifs, which means that the bond numbers have important roles when all variables are considered together.

## 4. Discussion

We compared random and natural non-coding RNA secondary structures (SSs), arriving at two main conclusions: Firstly, agreeing with and extending earlier works, we showed that natural and random RNA abstract shapes are overall very similar for lengths L≤400 nucleotides; structural motif counts (bulges, loops, helices, junctions, and bonds) are very similar for lengths L≤3000. Secondly, despite the overall similarity, we showed that the small differences in motif counts are sufficient for machine learning algorithms to classify natural and random RNA with good accuracy for larger RNA, which may be useful in detecting functional RNA in non-protein coding regions of the genome.

A major motivation for our work was to study the impact of GP map bias on evolutionary trajectories. By “bias” we mean that certain shapes have exponentially more sequences underlying them. Hence random mutations are far more likely to find such preferentially biased shapes. This bias is a known common property of many GP maps [55,56]. In this context, adding to earlier works, we suggest that our results here add weight to the case for GP map biases being substantial—if not actually dominant—players in determining the types of RNA shapes that exist in nature. Put differently, for the larger RNA we studied here, billions of possible shapes could appear in nature, but the action of the GP map bias restricts the shape repertoire very strongly, leaving the natural selection to tune and refine a much smaller set of possibilities. Even in light of earlier works, the overall close similarity of natural and random RNA is rather surprising, because the efficacy of functional RNA is largely determined by the shape, so, *a priori,* one would not expect them to (merely) be similar to random shapes.

Our work accords with experimental studies that have found that diverse structures with potential functionalities can be found in samples of random sequences [57,58], and reference [59], where it was shown that natural rRNAs have similar structural element properties (compared to random RNAs).

Our earlier findings that state that natural and random shapes are similar can be explained by the ‘arrival of the frequent’ theory proposed by Schaper and Louis [60], which states that even though selection acts on variation in a population, the GP map biases will strongly shape and constrain which types of variations appear for selection to act on. In their mathematical–computational study, it was shown that even in the presence of natural selection, phenotype bias can still dominate outcomes. See also references [29,61] for related computational studies and conclusions using RNA and a multi-level GP map. Our results also correlate with many other studies that found that bias can have a strong role in steering evolutionary trajectories [7,39,62,63,64,65,66,67], including the effects of mutation bias [12,68]. These works support the idea that non-isotropic variation is a significant factor in understanding evolution [69]. Relatedly, it has been shown that the ease of evolutionary accessibility, not relative functionality, can shape which gene network motifs evolve in nature [70].

Another possible explanatory factor for the similarity between natural and random SS is that some fitness-related properties of phenotype shapes are linked to bias. Recently, it has been mathematically argued that certain generic fitness requirements based on physics and engineering principles (e.g., mutational robustness in molecules and efficiency in biological networks) may lead to highly optimal values for particular types of phenotype shapes, which may also have high probability or be favorably biased [71,72]. In addition to mathematical arguments, a large range of biological examples is presented in support of the theory. Thus, it is possible that not only does GP map bias shape the variation presented to the selection, but there may also be a fitness preference for these shapes. Regardless of which of these explanations or combination of explanations is valid, it remains an interesting theoretical biology observation that the RNA shapes that appear in nature, and their frequencies, can be predicted by computational and physics-based reasoning.

From a completely different angle, it may be countered that natural selection has adapted RNA SS over time so that the folding rules are tweaked in order to make the types of RNA that are needed by organisms ‘easy’ to generate (rather than bias-shaping the types of RNA seen in nature, the types of RNA in nature shape the bias). This would explain the similarity of random and natural RNA from a purely selection-based argument. This proposal seems quite unlikely to be valid because RNA-folding rules are primarily based on chemistry and physics, so it is hard to see how selection could have much impact on these rules. Additionally, it has been shown that bias (and probability) are closely related to the information content, the complexity of shapes, and a general mathematical property [39,73,74]. Again, information content is not something that selection can substantially alter. See also reference [75] for the mathematical treatment of neutral set sizes in RNA, again pointing to the fact that bias is related to fundamental mathematical properties of maps and, hence, something that is unlikely to be substantially altered by the selection.

It is known that a single strand of RNA can fold into more than one possible structure, and some strands even form different structures in vivo and in vitro [76]. Further, even if a given sequence has a minimum free energy SS, which dominates over other suboptimal SS, the sequence will assume a different SS in accordance with the Boltzmann distribution [40,77]. As is common practice in biology and bioinformatics—as well as in the vast majority of earlier RNA SS studies—here, we simplified the GP map by assuming that the minimum free energy SS predicted by the computational folding package is ‘the’ single phenotype. In reference [32], a brief analysis was made regarding how abstract shapes change if this Boltzmann distribution is incorporated. It was reported that while the dot-bracket SS will fluctuate between various suboptimal folds, the overall shape and, hence, abstracted shapes do not vary drastically. Hence, we do not expect the use of this simplifying assumption to qualitatively affect our conclusions. Nonetheless, this simplification forms a limitation of our work.

A limitation of our proposed method to detect functional RNA using SS motif counts is that we implicitly assumed knowledge of the length of the relevant functional RNA sequences, such that we compared, for example, length *L* = 1000 natural structures to *L* = 1000 random sequences. In practice, given a long non-protein coding region of a genome, we would not know in advance the relevant length to study. Therefore this should be addressed in a future study before directly applying our classification result to detecting functional RNA. Other limitations of the current study are that the SS prediction methods employed cannot handle pseudoknots; we ignored the role of single-stranded regions in stabilizing tertiary interactions through noncanonical base pairing, and we ignored kinetic co-transcriptional structure effects, which may imply that the minimum free energy structure is different from the structure found in nature [78].

Research on proteins has shown that while the natural protein sequence space is vast, the number of corresponding protein folds is much smaller, estimated to be a few thousand at most [79,80]. Moreover, a small number of protein folds make up a significant fraction of the folds present in genomes. This observation is similar to the observation that the spaces of RNA structures and shapes sare much smaller than the spaces of sequences, and there is a kind of bias for certain shapes [25]. However, it is essential to note that we are not suggesting in this work that there are only a fixed (small) number of RNA shapes in nature. Earlier computational simulations [60] have demonstrated that more significant samples lead to more unique RNA shapes, but the rate of growth for unfound shapes is sluggish. This slow growth is due to the fact that different RNA shapes have probabilities that differ by orders of magnitude, and the expected number of samples needed to find a shape with probability *q* is 1/q. Additionally, as more natural RNA sequences are deposited into bioinformatic databases, we anticipate that the number of unique shapes will gradually increase (but at a slow rate relative to the number of added sequences).

While we focused on the sequence-to-shape map, the secondary structure shape is by no means the only important aspect of an RNA that is coded in the genome. Instead, there are many possible sequence-to-phenotype maps that may be studied in relation to RNA, such as the sequence-to-catalytic function, among others. Studying these other maps would be interesting for future work. Returning to the question of bias, in future work, it would be interesting to incorporate different structure prediction methods [81], especially RNA tertiary structure prediction, if and when it becomes available [82]. Furthermore, it would be interesting to study the interplay between bias and selection [66,83], whether natural or artificial (for example, investigating if the ‘arrival of the frequent’ can be observed experimentally). Possible ways this could be implemented include experimentally via estimating the fitness of RNA molecules [84,85,86], or via experimentation combined with deep learning methods to elucidate fitness landscapes, as has been done recently for RNA ligase ribozymes [87]. Such experiments would add data points where more concrete conclusions could be drawn regarding the role of bias in evolution.   

## Figures and Tables

**Table 1:** Linear fits m=aL+b for the number *m* of bulges, loops, junctions, helices, and bonds, for natural and random samples.

Motif	Natural	Random Samples
Bulges	0.010L−0.65	0.013L−0.12
Loops	0.020L+0.94	0.018L+0.65
Junctions	0.0083L−0.60	0.0085L−0.51
Helices	0.070L+1.78	0.073L−0.049
Bonds	0.31L+2.1	0.32L+5.8

**Table 2:** The 95% confidence intervals for the linear fit parameters *a* (slope) and *b* (intercept) given in Table 1.

(a) Slopes		
**Motif**	**Natural**	**Random Samples**
Bulges	[0.010, 0.011]	[0.012, 0.013]
Loops	[0.011, 0.020]	[0.012, 0.018]
Junctions	[0.0082, 0.020]	[0.0084, 0.018]
Helices	[0.0082, 0.070]	[0.0084, 0.073]
Bonds	[0.0082, 0.32]	[0.0085, 0.32]
**(b) Intercepts**		
**Motif**	**Natural**	**Random Samples**
Bulges	[−0.97, −0.35]	[−0.50, 0.28]
Loops	[−0.90, 1.2]	[−0.42, 0.86]
Junctions	[−0.85, 1.2]	[−0.62, 0.82]
Helices	[−0.81, 2.1]	[−0.61, 0.79]
Bonds	[−0.79, 2.6]	[−6.4, 0.76]]

**Table 3:** ROC AUC values and the 95% confidence interval values for lengths *L* = 100, 400, 1000, and 1000 with adjusted GC content (‘scrambled’ natural RNA). Table (a) uses kNN and (b) uses PLSDA.

(a) kNN		
**Length (*L*)**	**Original ROC Area**	**95% Confidence Interval**
100	0.72	[0.72–0.73]
400	0.81	[0.81–0.82]
1000	0.86	[0.85–0.88]
1000 GC adjusted	0.86	[0.85–0.87]
**(b) PLSDA**		
**Length (*L*)**	**Original ROC Area**	**95% Confidence Interval**
100	0.70	[0.70–0.71]
400	0.78	[0.78–0.78]
1000	0.83	[0.82–0.84]
1000 GC adjusted	0.83	[0.82–0.84]

**Figure A2:**
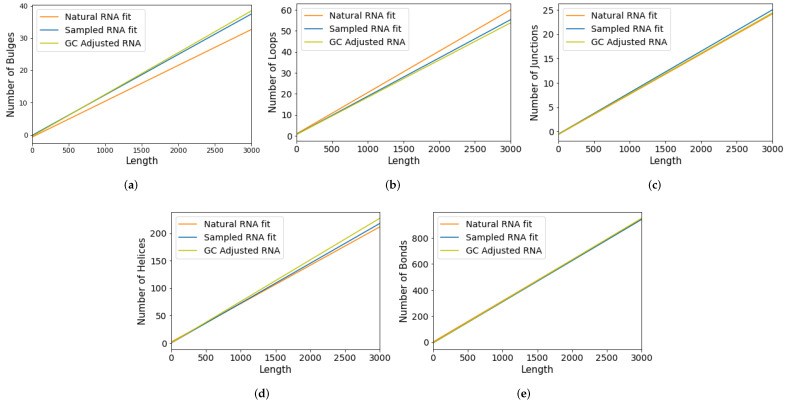
Fits comparing the natural and random RNA, and GC adjusted by scrambling the sequences. GC content does not make a large difference in the scaling of motif counts with *L*, as can be inferred from the fact that the blue and green lines have similar slopes and intercepts.

## Data Availability

The data and code used in this work are available at https://github.com/fatmeghaddar/RNA-Motif-Patterns.git.

## Supplementary Materials

The following supporting information can be downloaded at: https://www.mdpi.com/article/10.3390/life13030708/s1: Data sheet containing information about common RNA shapes, and data sheet containing information about the similarity of shapes with similar motif counts.

## Appendix A. Methods

### Appendix A.1. Code and Data Availability

The code and data used in this work are available from https://github.com/fatmeghaddar/RNA-Motif-Patterns.git.

### Appendix A.2. Random RNA Sequences

We created random sequences using Python, with a uniform probability of 0.25 for each nucleotide A, U, C, G. The random sample numbers were $3\times 10^{4}$ for L=100, L=200, L=300, and L=400 for Figure 2 and Figure 3. For the linear fits to motif counts depicted in Figure 4, the lengths L=50, 100, 150, 200, 250, ⋯, 3000 were used, and for each length, 20 random sequences were made.

### Appendix A.3. Natural RNA Sequences

All natural sequences of lengths L=50,100,150,200,250,⋯,3000 were downloaded from the RNAcentral database [44] and underwent a cleaning process where any repeated sequences were removed. Any sequences that contained non-standard nucleotide letters were excluded from the samples. For Figure 2 and Figure 3, all acceptable sequences were used. For Figure 4 and the linear fits, because the use of the complete RNAcentral dataset of the specified lengths resulted in a large 4 GB file, analyzing this dataset would be computationally taxing. Further, because we only required these natural samples to make linear fits, it is not necessary to have such a large dataset to work with. Hence, random sub-sampling was applied to the dataset, where only 20 randomly selected natural sequences of each length were used for the linear fits.

### Appendix A.4. Folding RNA

We used the popular RNA Vienna package [43] to predict SS from the sequences. We set all parameters to their default values (e.g., the temperature *T* = 37 °C).

### Appendix A.5. Drawing RNA

Part of Figure 1 was made by drawing an RNA SS using the online tool http://rna.tbi.univie.ac.at/forna/, accessed on 15 December 2022.

### Appendix A.6. Motif Counting

To count the structural motifs, i.e., the number of bonds, helices, loops, bulges, and junctions on each SS, we used the Python code Secstruc.py (written by K. Rother). This code is available as a file in the Supplementary Information, along with other codes for this project.

### Appendix A.7. Abstract Shapes

Similar to our previous work [32], coarse-grained abstract shapes were used where RNA SS were abstracted from standard dot-bracket notations in different levels. The abstract shapes were obtained using the RNAshapes tool available at bibiserv.cebitec.uni-bielefeld.de/rnashapes and the Bioconda rnashapes package available at anaconda.org/bioconda/rnashapes. In order to accommodate the Vienna folded structures, the option to allow single-bonded pairs was chosen. In this present work, we used abstract shape level 5, in which no unpaired regions were considered (only if the entire structure was unpaired), and nested helices were combined. Figure 1 in the main text illustrates the abstraction process.

## Appendix B. Motif Counts and Overall RNA Shape

Because the spaces of possible abstract RNA shapes were constrained by the motif counts (i.e., the number of bulges, loops, junctions, helices, and bonds), we can expect that two RNAs with the same motif counts will have somewhat similar abstract shapes. To investigate the connection between motif count similarity and shape similarity, we took the L=100 data and grouped together all RNA into classes with the same motif counts (e.g., all RNA with 1 bulge, 1 loop, 0 junctions, 7 helices, and 26 bonds classed together). We then counted the number of different shapes adopted for each class and found that typically around 10 different shapes occurred. Hence, motif count similarity does not directly equate to shape similarity.

Looking at this question more closely, while there are roughly 10 shapes per class that appeared, these shapes do not each appear with equal probability, and some of the shapes were very rare. To give a concrete example, there were 155 sequences in the class of RNA with motif counts as follows: 1 bulge, 1 loop, 0 junctions, 7 helices, and 26 bonds. These 155 RNA were distributed between the corresponding shapes as follows: ‘[][]’: 57, ‘[[][]]’: 26, ‘[][][]’: 22, ‘[]’: 21, ‘[[][][]]’: 11, ‘[][][][]’: 6, ‘[[][]][]’: 5, ‘[[][][][]]’: 3, ‘[][[][]]’: 2, ‘[][[][][]]’: 1, ‘[[][][]][]’: 1.

From this, we see that while there are 11 shapes in this class, several of them appeared only rarely. Hence, the ‘effective number’ of shapes in the class is less than 11. To quantify this effective number, we used the standard method of $2^{H}$, where *H* is the Shannon entropy (in bits) of the distribution. The value $2^{H}$ will be roughly equal to the actual number of shapes (states) if the probability distribution over shapes is roughly uniform. On the other hand, if only a few shapes account for almost all of the probability, then $2^{H}$ will be roughly equal to this effective number of states. Computing $2^{H}$ for L=100 data, we find that the average was only 3.1, which is substantially less than ∼10. In conclusion, for this dataset, the identical motif counts do not equate to identical shapes; on the other hand, the effective number of shapes is quite small.

See the Supplementary Information File L100_Random_Motifs_Shapes_Shannons Entropy.csv for the motif counts and entropy values.

## Appendix C. Adjusting for GC Content

In Figure A2, we show linear fits for motif frequencies where instead of purely random sequences, randomly permuted (‘scrambled’) natural sequences are used. As is apparent, adjusting for GC content does not make a substantial difference to motif counts.
